# Does Naloxone Reinstate Secondary Hyperalgesia in Humans after Resolution of a Burn Injury? A Placebo-Controlled, Double-Blind, Randomized, Cross-Over Study

**DOI:** 10.1371/journal.pone.0064608

**Published:** 2013-05-31

**Authors:** Manuel P. Pereira, Mads U. Werner, Thomas K. Ringsted, Michael C. Rowbotham, Bradley K. Taylor, Joergen B. Dahl

**Affiliations:** 1 Multidisciplinary Pain Center, Neuroscience Center, Rigshospitalet, Copenhagen University Hospitals, Copenhagen, Denmark; 2 Department of Anaesthesia, Centre of Head and Orthopaedics, Copenhagen University Hospitals, Rigshospitalet, Copenhagen, Denmark; 3 California Pacific Medical Center Research Institute, San Francisco, California, United States of America; 4 Department of Physiology, University of Kentucky Medical Center, Lexington, Kentucky, United States of America; University of Arizona, United States of America

## Abstract

**Introduction:**

Development of secondary hyperalgesia following a cutaneous injury is a centrally mediated, robust phenomenon. The pathophysiological role of endogenous opioid signalling to the development of hyperalgesia is unclear. Recent animal studies, carried out after the resolution of inflammatory pain, have demonstrated reinstatement of tactile hypersensitivity following administration of μ-opioid-receptor-antagonists. In the present study in humans, we analyzed the effect of naloxone when given after the resolution of secondary hyperalgesia following a first-degree burn injury.

**Methods:**

Twenty-two healthy volunteers were included in this placebo-controlled, randomized, double-blind, cross-over study. Following baseline assessment of thermal and mechanical thresholds, a first-degree burn injury (BI; 47°C, 7 minutes, thermode area 12.5 cm^2^) was induced on the lower leg. Secondary hyperalgesia areas around the BI-area, and separately produced by brief thermal sensitization on the contralateral thigh (BTS; 45°C, 3 minutes, area 12.5 cm^2^), were assessed using a polyamide monofilament at pre-BI and 1, 2, and 3 hours post-BI. At 72 hrs, BI and BTS secondary hyperalgesia areas were assessed prior to start of a 30 minutes intravenous infusion of naloxone (total dose 21 microg/kg) or placebo. Fifteen minutes after start of the infusion, BI and BTS secondary hyperalgesia areas were reassessed, along with mechanical and thermal thresholds.

**Results:**

Secondary hyperalgesia areas were demonstrable in all volunteers 1–3 hrs post-BI, but were not demonstrable at 72 hrs post-burn in 73–86% of the subjects. Neither magnitude of secondary hyperalgesia areas nor the mechanical and thermal thresholds were associated with naloxone-treated compared to placebo-treated subjects.

**Conclusion:**

Naloxone (21 microg/kg) did not reinstate secondary hyperalgesia when administered 72 hours after a first-degree burn injury and did not increase BTS-generated hyperalgesia. The negative results may be due to the low dose of naloxone or insufficient tissue injury to generate latent sensitization.

## Introduction

Considerable research effort has been invested in examining the contribution of central sensitization [1] to development of chronic pain [2]–[7]. In chronic pain conditions such as neuropathic pain, fibromyalgia or chronic tension headache, the endogenous opioid modulation of central sensitization is impaired or altered [2], [8]–[12].

In experimental research in rodents, injury or exposure to opioid may produce long-lasting vulnerability, termed latent sensitization [13], to noxious stimuli [14], [15], non-noxious environmental stress stimuli [13], [16], ultralow doses of opioid [16] and opioid antagonists [17]–[19]. Administration of naloxone and naltrexone to animals, following resolution of an inflammatory injury, has demonstrated a NMDA-receptor dependent re-instatement of hypersensitivity to noxious stimuli near or at the injured area [16]–[19]. It has been hypothesized that the endogenous opioid-dependent mechanisms are responsible for the transition from acute to chronic pain in humans [13], [16], [17]. Translational research, from animals to humans, in latent sensitization is of critical importance, since insight in these pathological mechanisms may lead to reformulation of strategies for prevention of chronic pain.

A number of human sensitization models using capsaicin [20], electrical stimulation [8], and thermal injury [2], have been used to evaluate secondary hyperalgesia (i.e. hyperalgesia or allodynia in normal skin surrounding the injury site), a centrally mediated event [21], [22]. Development of secondary hyperalgesia is modulated by various drugs: adenosine [23], gabapentin [24], glucocorticoids [25], NMDAR (*N*-Methyl-D-aspartate-receptor) blockers [26], [27], and opioids [28]–[30]. However, the effects of naloxone *per se* on secondary hyperalgesia areas are more ambiguous [2], [8], [31]–[33].

In the present study, we used a first-degree burn injury (BI) as a validated inflammatory model of sensitization [34], [35]. The primary aim was to examine if naloxone could re-instate secondary hyperalgesia areas after resolution of the thermal injury. The secondary aim was to examine the effect of naloxone on secondary hyperalgesia areas produced by brief thermal sensitization on the contralateral thigh (BTS) and, on thermal and mechanical thresholds in the primary hyperalgesia area.

## Methods

### Volunteers

The study protocol was approved by The Committees on Health Research of the Capital Region of Denmark and the Danish Medicines Agency (Protocol no.: H-2-2012-036, EudraCT nr.: 2012-000839-54). The study was conducted according to the principles of Good Clinical Practice (GCP), and monitored by the Copenhagen University Hospitals’ GCP-unit. Healthy volunteers were recruited to participate in this study through flyers and advertisements at campuses at Copenhagen University, or from own records from completed studies. Twenty three volunteers were screened for eligibility. Inclusion and exclusion criteria are presented in Table 1. All volunteers were provided information regarding the study and its possible risks and signed a written consent. The volunteers were paid EUR 300 (USD 385) as a compensation for their participation in the study.

**Table 1:** Inclusion and exclusion criteria.

Inclusion Criteria	Exclusion Criteria
• ASA I-II	• not cooperative
• 20≤ age ≤35 years	• not understand or speak Danish or English
• urine sampled negative for amphetamines, barbiturates, benzodiazepines, cocaine, opioids (buprenorphine, methadone, morphine) and tetra-hydrocannabinol (THC)	• pregnancy, breastfeeding, planning pregnancy or who were not using contraceptives(pill or IUD)
• 18 kg/m^2^< BMI and <30 kg/m^2^	• participated in a drug trial in the previous 60 days
	• alcohol or drug abuse
	• use of psycho-active drugs or analgesics
	• neurological illness
	• chronic pain condition
	• allergy to morphine or naloxone
	• skin lesions on the measurement areas
	• signs of a neuropathy in the ipsilateral or contralateral measurement areas
	• prescription drugs 1 week before the trial
	• over-the–counter medication 48 hours before the test

### Study Design

The study followed a placebo-controlled, double-blind, randomized, cross-over design.

### Study Algorithm

**Figure 1:**
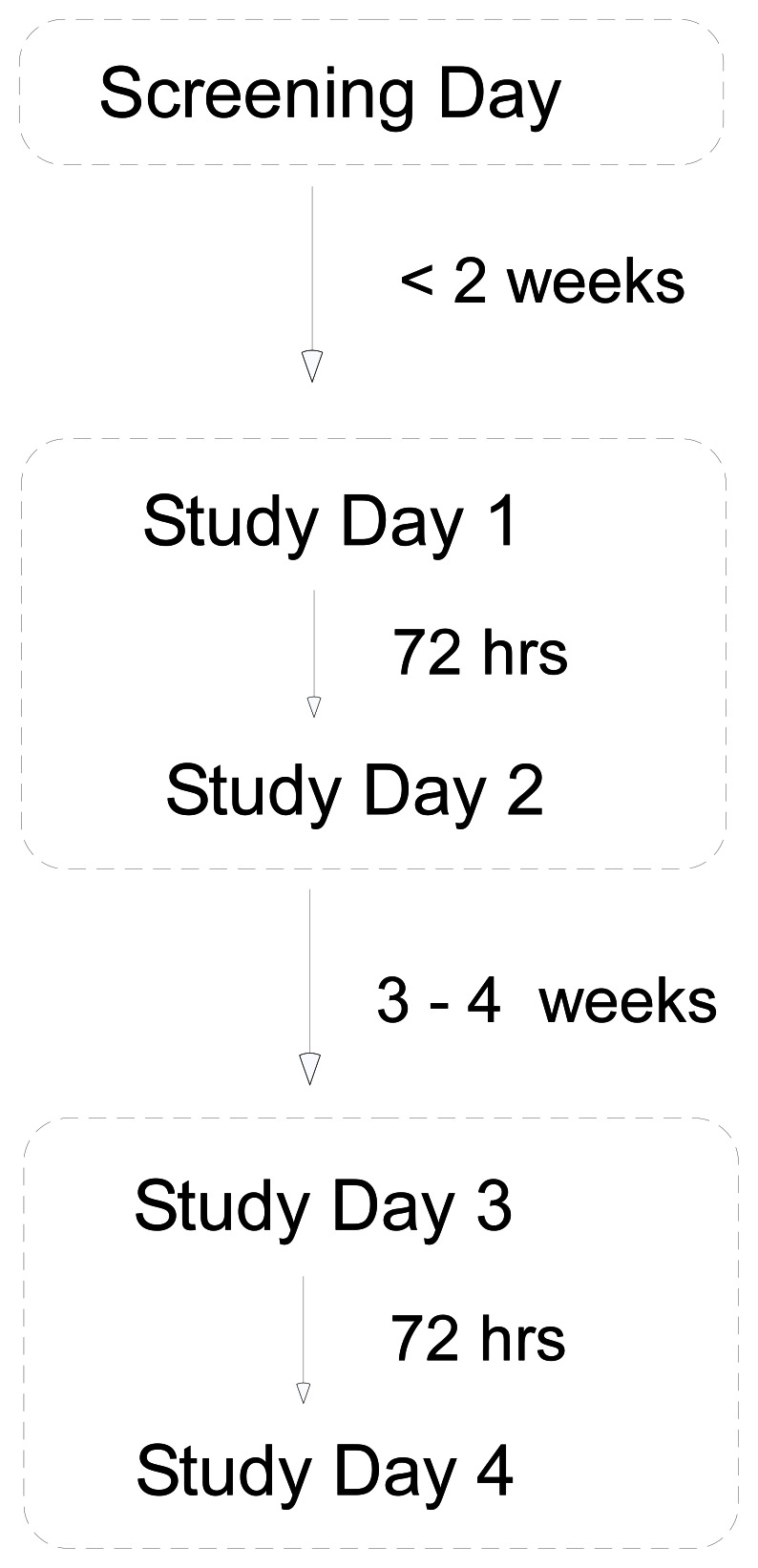
Study algorithm. The study was performed on 5 separate days. Day 0 corresponded to the screening day; Day 1 and Day 3 were the burn injury days separated by 72 hrs from the drug administration days, Day 2 and Day 4.

**Figure 2:**
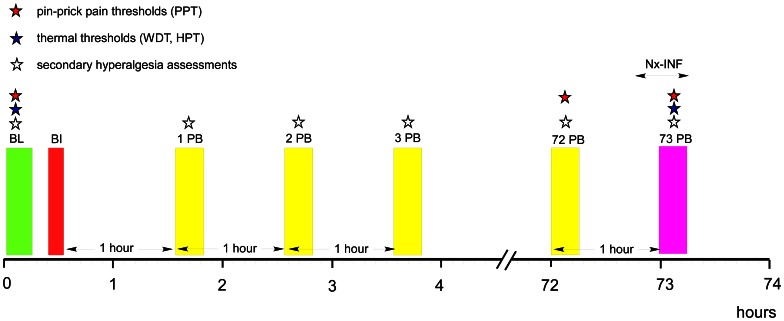
Detailed timetable algorithm of the study. (Study Days 1 and 2, and, Study Days 3 and 4 are identical). BL = baseline (warmth detection thresholds, heat pain thresholds, pinprick pain thresholds, secondary hyperalgesia areas in brief thermal stimulation and burn injury sites), Nx-INF = Naloxone target-controlled infusion (see text for detailed explanation). 1/2/3 PB = postburn assessments 1, 2 and 3 hrs after the burn injury (secondary hyperalgesia areas on brief thermal stimulation and burn injury sites), 72 PB = postburn assessments 72 hrs after the burn injury (pinprick pain thresholds, secondary hyperalgesia areas on brief thermal stimulation and burn injury sites), 73 PB = postburn assessments 73 hrs after the burn injury (warmth detection thresholds, heat pain thresholds, pinprick pain thresholds, secondary hyperalgesia areas on brief thermal stimulation and burn injury sites).

The study was performed on 5 separate days (Figs. 1 and 2). On Day 0 volunteers were screened whether they were eligible to participate in the study and they were familiarized with assessments and the BI on their dominant leg. Day 1 and Day 3 were the BI-days separated by 72 hrs from Day 2 and Day 4 which were the drug administration days. Between Day 1 and Day 3 there was a wash-out period of 3–4 weeks. If volunteers received naloxone on Day 2, they would then get placebo on Day 4 and *vice-versa*.

### Randomization Procedure

The randomization procedure was performed by a research nurse not participating in the study. A total of 28 subject-numbers were randomly allocated into two groups (A and B) using a randomization software (randomisation.com). Groups A and B were randomized to start with either naloxone or placebo. For each subject-number the randomization code was included in a non-transparent envelope. The envelope and 6 ampoules of naloxone 0.4 mg/ml (Naloxon "B. Braun", B. Braun Melsungen, Germany), were packed for each subject-number in an opaque sealed box.

The infusions were prepared up to 8 hrs before the study session by a research nurse or physician, not participating in the study and not employed in the department. The naloxone infusion was prepared by diluting 6 ml of naloxone 0.4 mg/ml with 154 ml normal saline, obtaining a concentration of naloxone 15 microg/ml. The placebo infusion was normal saline in an identical volume, 160 ml. The individual randomization codes for each session were returned into the respective envelope, and empty or unused ampoules were returned to the box, which then was resealed. There was no contact between the research nurse and the physician preparing the infusions, and, the investigators.

### Setting

#### Environment

The experimental procedures were performed in a quiet, bright room with a temperature 24–27°C and a relative humidity (RH) of 30–63%. The testing sessions were made between June 12^th^ and August 16^th^ 2012, and were carried out Mondays to Fridays between 07.30 AM and 08.00 PM. Subjects adopted a comfortable supine position during the assessments, and were allowed to move freely in adjacent rooms between the assessments.

#### Pin-prick thresholds

The area for quantitative sensory testing (QST) was the upper, medial part of the non-dominant lower leg. The subjects were instructed to use a hair-trimmer in the area, 2 days before the study days, in order to avoid interference with the sensory assessments. The rectangular BI area, 2.5×5.0 cm^2^, was delineated with the upper anterior corner 11 cm below the medial meniscus margin and 6 cm from the anterior margin of the tibia.

Pin-prick pain thresholds (PPT) were assessed by weighted-pin stimulators (PinPrick, MRC Systems, Heidelberg, Germany (8, 16, 32, 64, 128, 256, 512 mN)) with a contact-area of 0.31 mm^2^. Five assessments were performed according to Dixon’s “up-and-down” method [36]. Volunteers were stimulated 5 times at the site of the BI and were asked to indicate when ≥3 of the pin-pricks were perceived as painful. Using pin-prick stimulators of ascending or descending order, the PPT was determined 5 times and the median of these assessments was then considered for analysis. PPT assessments were performed at all study days.

#### Thermal thresholds

Warmth detection threshold (WDT) and heat pain threshold (HPT) were assessed in the BI area by a contact thermode (Thermotest, Somedic AB, Hörby, Sweden (12.5 cm^2^)), as previously described in detail [37]. The thresholds were determined from a baseline temperature of 32°C with a ramp rate of 1°C/s and 50°C was the cut-off temperature. The assessments were made in triplicate and the mean value was used in further analyses. Thermal thresholds were assessed at baseline and 73 hrs. after the BI (Fig. 2).

#### Burn injury

The first-degree BI was induced with a contact thermode (Thermotest, Somedic AB, Hörby, Sweden (12.5 cm^2^, 47.0°C, 7 minutes)). The pain intensity during the BI was rated on a visual analog scale (VAS (0 = no pain, 100 = maximum imaginable pain)) at 0, 30, 60, 120, 180, 240, 300, 360 and 420 seconds after the thermode had reached 47.0°C.

#### Brief thermal sensitization (BTS)

The application area was delineated on the skin, with the lower border of the rectangle 11 cm superior to the upper border of the patella in the mid line. A noxious tonic heat stimulus of 45°C was delivered to the anterior side of the dominant thigh using the contact thermode, as previously described [24], [38]. After a 180 s stimulation period, the area of secondary hyperalgesia was assessed using a polyamide monofilament (nominal value 18 (890±50 mN (mean±SD)), Stoelting, IL, USA) [37] with the heated thermode *in situ*. Heat stimulation was limited to a maximum of 300 seconds. The BTS assessments were performed on Day 1 and Day 3 at baseline, and 1, 2, and 3 hours post-burn. On Day 2 and Day 4, assessments were made before and during the infusions at 72 and 73 hours post-burn (PB).

Secondary hyperalgesia areas were assessed using a polyamide monofilament (nominal value 18). The border was determined by stimulating in 8 symmetric lines each separated by an angle of 45° converging towards the centre of the burn injury. The stimulations started in normal skin outside the area of secondary hyperalgesia and the subjects, who had their eyes closed during the assessments, reported the occurrence of a definite change in sensation, to an uncomfortable, burning or stinging sensation. The corners of the octagon were marked on the skin and transferred to a transparent sheet. The secondary hyperalgesia areas were calculated (total area - area of the thermode) using a computer-based vector-algorithm (Canvas 12.0, ACD Systems International, Victoria, Canada).

Assessments of secondary hyperalgesia areas on Day 1 and Day 3 were done at baseline, and, 1, 2 and 3 hours PB. On Day 2 and Day 4 assessments of secondary hyperalgesia areas were made before the infusions at 72 hours PB and during the infusions at 73 hours PB.

### Drugs

On Day 2 and Day 4 (Figs. 1 and 2) a 30 minutes intravenous, target-controlled infusion of naloxone 15 microg/ml or placebo was administered starting 72 hrs 45 minutes after the BI [8]. An i.v. bolus of naloxone was administered (5 microg/kg) during 2 minutes, followed by an infusion at rate of 40 microg/kg/h for 20 minutes and finally, at a rate of 20 microg/kg/h for 8 minutes [8]. Thus, volunteers were given a total 21 microg/kg of naloxone over 30 minutes. Identical administration volumes (1.4 ml/kg) and algorithm was used for placebo-infusion [8].

### Statistics

Estimating the sample size, a significance level of 0.01 (α), a power of 0.9 (β = 0.1), an intra-individual standard deviation (SD) of secondary hyperalgesia areas at 72 hrs after the burn injury, of 5 cm^2^, and a minimal relevant difference 5 cm^2^ were used. Since no data are available in regard to these estimates,(this is the first study in this area) we used estimates that were considered relevant for the sample size calculation. Under the assumptions that data would be normally distributed and that the study had a cross-over design, the estimated number of subjects needed were 19. However, in order to compensate for any drop-outs, the number of volunteers was set to 22.

To test if data was normally distributed, the Kolmogorov-Smirnov test and residual plots were used. In case of non-normal distribution, a logarithmic transformation was tried for normalization of the data. Paired *t*–test was used for comparison in case of normally distributed data, whereas Wilcoxon rank sum test was used for non-normally distributed data. Fisher’s exact test was used in the analysis 2×2 contingency tables. A *P*-value of 0.01 was assigned as the significance level.

After completion of the study, data was first partially unblinded for statistical analyses: subjects were divided into group A and B (see Randomization Procedure). Only after completion of the statistical analyses, data were fully unblinded.

Data are given as mean (SD) or median (25–75% interquartile range [IQR]).

## Results

### Demographic Data

A total of 23 volunteers were included in the study. However, one volunteer (#4) was excluded, due to participation in another study less than 60 days before. Thus, per-protocol data from 22 healthy volunteers (11 females, 11 males) were included in the present study. Demographic data are illustrated in Table 2.

**Table 2:** Demographic data.

	n	Age (yrs)	Height (cm)	Weight (kg)
Male	11	24.5±2.0	181.3±3.3	77.7±6.9
Female	11	23.0±1.2	172.2±5.0	66.7±6.4

Mean values±SD.

### The Burn Injury

#### Pain during induction

The volunteers described mild to moderate pain during the 7 minutes burn with VAS/minute -ratings Day 1∶30.4±2.3 and Day 2∶28.8±1.5. No statistically significant habituation effect, i.e. decrease of perceived pain intensity throughout the study days, was observed between Day 1 and Day 3 (*P* = 0.21 [Table 3]).

**Table 3:** Cumulative VAS scores (0–100).

	Day 1		Day 2	
	Cumulative VAS	VAS/minute ± SD	Cumulative VAS	VAS/minute ± SD
Burn-injury	5348	30.4±2.30	–	–
BTS	767	8.7±1.47	462	5.3±0.88
	Day 3		Day 4	
Burn-injury	5068	28.8±1.47	–	–
BTS	627	7.2±0.99	462	5.3±0.23

VAS/minute and standard deviation reported by the volunteers during the burn injury (Day 1+3) and BTS (Day 1+2+3+4). No difference in cumulative VAS was observed between Day 1 and 3 during the burn injury (*P = *0.21) and during BTS (*P = *0.09). There was a significant difference between Day 1 and 2 (*P*<0.01), and Day 3 and 4 in VAS ratings during BTS (P<0.05). BTS = Brief thermal stimulation.

#### Local skin changes

Erythema and hyperalgesia were seen in all volunteers following the BI. No residual effects related to the BI were observed, with the exception of one volunteer (#14), who developed small areas of hyperpigmentation at the injury-site 23 days after the BI. No blisters were observed.

#### Secondary hyperalgesia areas

**Figure 3:**
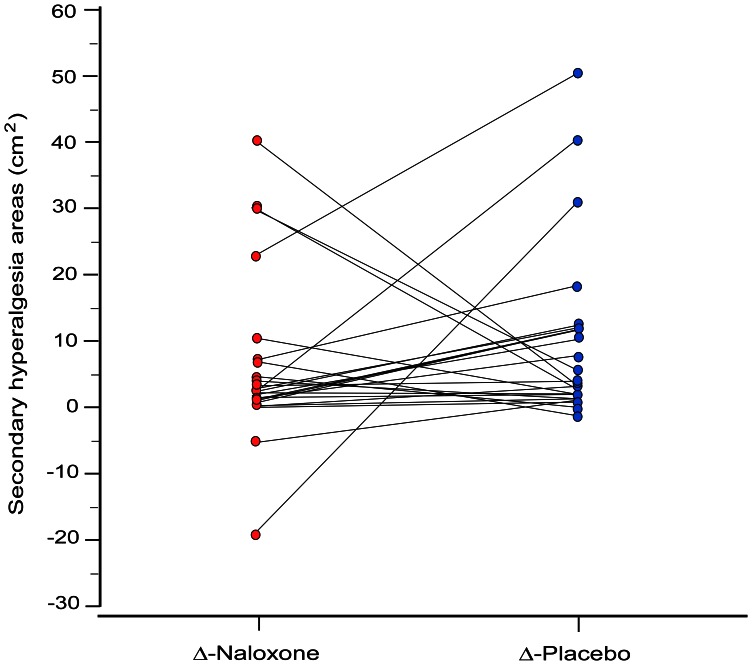
Size of secondary hyperalgesia areas after naloxone or placebo administration. Individual secondary hyperalgesia areas (▵-values = post-infusion area – pre-infusion area) at burn injury site in cm^2^ after administration of naloxone and placebo, 72 hrs post-burn. The median (25–75% interquartile range) change in secondary hyperalgesia areas after naloxone administration was 1.87 cm^2^ (0.74–7.00) and after placebo administration 3.10 cm^2^ (1.48–11.42). Magnitude of secondary hyperalgesia areas was not associated with naloxone-treated compared to placebo-treated subjects (*P = *0.25).

Secondary hyperalgesia areas were observed in all volunteers, in both baseline assessment days (Day 1 and 3), with the exception of one volunteer (#12), who did not develop a measurable area in one of the days (Day 3). Secondary hyperalgesia areas were significantly larger on Day 1 compared to Day 3 (*P*<0.01), indicating a habituation effect. On Days 2 and 4, three volunteers had detectable secondary hyperalgesia areas before infusion of naloxone, and 6 volunteers before infusion of placebo (*P* = 0.46). Nine volunteers developed larger areas of secondary hyperalgesia after receiving naloxone compared to placebo, whereas 13 volunteers developed (larger) secondary hyperalgesia areas after placebo infusion compared to naloxone [Fig. 3]). However, when comparing both distributions - which is the primary endpoint of this study - there were no significant changes in the magnitude of hyperalgesia areas following naloxone or placebo (*P = *0.25).

There was an agreement between BI and BTS data (below) in regard to changes in secondary hyperalgesia areas with administration of naloxone when compared to placebo: the sign-test showed that 16 volunteers had congruent findings with both methods, while 6 volunteers had different findings.

### Brief Thermal Stimulation

#### Pain during induction

The BTS procedure induced only a mild pain, with low VAS-ratings (VAS/minute±SD) of 8.7±1.5 (Day 1), 5.3±0.9 (Day 2), 7.2±1.0 (Day 3) and 5.3±0.23 (Day 4), (Table 3). Pain assessments were performed at baseline on Days 1 and 3, and then again 72 hours post-burn on Days 2 and 4. No statistically significant habituation effect was evident between Days 1 and 3 (*P* = 0.09). However, there was a significant habituation effect between Day 1 and Day 2 (*P*<0.01) with lower values on Day 2. A similar effect was seen between Day 3 and Day 4 (*P*<0.05) [39].

#### Local skin changes

Erythema and hyperalgesia were seen in all volunteers following BTS. No blisters or other residual effects were observed.

#### Secondary hyperalgesia areas

Development of secondary hyperalgesia areas was observed in all volunteers following BTS. Administration of naloxone was not associated with a change the areas of the secondary hyperalgesia compared to placebo (*P = *0.76). Nine volunteers developed larger areas after infusion of naloxone when compared to placebo, while 13 volunteers developed larger areas after infusion of placebo compared to naloxone (Fisher’s exact test, *P* = 0.37).

There was an interval of 23.0±2.2 days between Day 1 and Day 3. This interval was associated with a habituation in induction of hyperalgesia areas, i.e. significantly larger areas on Day 1 were observed when compared to Day 3 (*P*<0.01) [39].

### Mechanical Thresholds

The PPT, assessed in the BI-area, did not change with administration of naloxone when compared to placebo (*P = *0.98, [Table 4]).

**Table 4:** WDT, WDT and PPT.

	Day 1	Day 2		
		Pre-Inf	Post-Inf	▵(Day 2_post_–Day1)
WDT (°C)	4.41±1.56	–	4.80±1.64	0.40±1.39 (*P = *0.10)
HPT (°C)	44.59±2.34	–	44.02±2.50	−0.57±2.37 (*P = *0.27)
PPT (mN)	512 [512;513]	512 [256;512]	512 [128;512]	–
	**Day 3**	**Day 4**		
		**Pre-Inf**	**Post-Inf**	**▵(Day 4_post_ -Day3)**
WDT (°C)	4.89±2.11	–	5.11±2.23	0.23±1.58 (*P = *0.13)
HPT (°C)	44.90±2.19	–	44.49±2.56	−0.41±1.61 (*P = *0.12)
PPT (mN)	512 [256;513]	512 [256;512]	256 [256;512]	–

Mean value and standard deviation of WDT and HPT are shown in this table, as well as median values and 25–75% IQR of PPT. On Day 2 and 4, pin-prick assessments were performed before and after i.v. administration of naloxone or placebo, whereas HPT and WDT were only assessed after drug infusion. Naloxone administration was not associated with changes in WDT (*P = *0.39), HPT (*P = *0.21) and PPT (*P = *0.98). There were no significant differences in WDT and HPT, assessed in the BI-area, between Day 1 and 2 ([baseline *vs.* 73 hrs PB, Fig. 2] *P = *0.10, *P = *0.27, respectively), and between Day 3 and 4 (*P = *0.13, *P = *0.12, respectively).

HPT = Heat pain thresholds, PPT = Pin-prick thresholds, WDT = Warmth detection thresholds.

### Thermal Thresholds

There were no significant differences in WDT and HPT, assessed in the BI-area, between Day 1 and 2 ([baseline *vs.* 73 hrs PB, Fig. 2] *P = *0.10, *P = *0.27, respectively), and between Day 3 and 4 (*P = *0.13, *P = *0.12, respectively [Table 4]). Naloxone administration was not associated with changes in WDT (*P = *0.39) or HPT (*P = *0.21), when compared to placebo.

### Adverse Drug-related Effects

No drug-related adverse effects were observed in this study.

## Discussion

In the present placebo-controlled, crossover study in humans, we were not able to demonstrate naloxone-mediated reinstatement of secondary hyperalgesia areas following resolution of a first-degree thermal burn injury (BI). Naloxone changed neither secondary hyperalgesia produced by BTS nor mechanical or thermal thresholds in the primary hyperalgesia area. There are several possible reasons why the present study in humans did not produce the same results as earlier studies in rodents. *First*, the dose of naloxone may have been too low. *Second*, the superficial thermal injury, producing only limited tissue injury, may have been deficient for generating latent sensitization. *Third*, the time point chosen for looking for latent sensitization may have been incorrect. In animals, the situation is different with naloxone robustly reinstating secondary hyperalgesia long after a primary injury has apparently healed. *Fourth*, methodological inadequacies may have been present. *Fifth*, species differences may be such that the phenomenon has a different underlying mechanism or is expressed differently.

### Mechanisms of Latent Sensitization in Animals

Intraplantar injection of complete Freund’s adjuvant (CFA) in mice produces mechanical hypersensitivity, evidenced by a reduction in tactile thresholds [18], [19]. Following complete resolution of the hypersensitivity, 21 days after the injury, intrathecal or systemic administration of naltrexone or CTAP (MOR-selective antagonist), is associated with reinstatement of mechanical hypersensitivity [19]. Intrathecal administration of pertussis toxin, destroying G-α-subunit (Gα_i/o_)-proteins, also leads to a reinstatement of mechanical hypersensitivity, suggesting a tonic activity of inhibitory GPCRs (G Protein-Coupled Receptors) signalling [18], [19]. Pre-treatment with MK-801 (Dizocilpine), a non-competitive NMDAR-blocker, prevents the reinstatement of mechanical hypersensitivity, indicating that latent pain sensitization is dependent on NMDAR activity [18], [19]. These studies suggest that NMDAR-activity regulates a form of spinal sensitization that persists long after the resolution of inflammatory hyperalgesia. An up-regulated, tonic activation of opioid receptors, functionally coupled to Gα_i/o_-proteins, prevents this spinal sensitization from remaining clinically apparent until an opioid receptor blocking agent is administered.

### Naloxone Dose

The effective systemic doses of opioid antagonists used in animal studies to demonstrate latent sensitization have been 1 mg/kg of naloxone [17] or 0.3 to 3.0 mg/kg of naltrexone (unpublished studies, Taylor BK). Estimates of equipotency of naltrexone and naloxone depend on route of administration and the pharmacodynamic efficacy measure: antagonism of opioid analgesia, reversal of opioid-induced ventilatory depression, precipitation of withdrawal symptoms or inhibition of discriminative effects of opioids [40]. The available estimates from animal studies indicate a 2–4 higher potency for systemically administered naltrexone compared to naloxone [41], [42]. The dose of naloxone 0.021 mg/kg used in the present study is much lower than in the animal studies, and could therefore explain our failure to demonstrate latent sensitization.

In the present study, a target-controlled infusion, corresponding to an estimated plasma naloxone concentration of 10 ng/mL, was used. This dose regimen is identical to a study with intradermal, high current-density electrical stimulation, which demonstrated significant increases in established secondary hyperalgesia area following naloxone administration [8]. However, the high current-density stimulation is administered over a longer time period and is both more painful than BI and BTS and persists as long as the electrical stimulation continues. The increased magnitude of established secondary hyperalgesia, during ongoing electrical stimulation, by administering naloxone, is evidence that the inhibitory endogenous opioid system is immediately activated and thus not analogous to the experimental paradigm used in the present study.

A number of human hyperalgesia studies [2], [32], [33], [43] have used higher doses of naloxone, up to 0.1 to 0.2 mg/kg, without demonstrating any hyperalgesic effects during other types of acute experimental pain. High doses of 1–2 mg/kg of naloxone have been used in clinical and experimental psychiatric, endocrinological, neurological or nutritional studies in patients [44]–[49] and in healthy volunteers [50]–[54]; however, this dose-range has not been used in human pain research. A Positron Emission Tomography study in volunteers with naloxone 0.1 mg/kg demonstrated a complete inhibition of the binding of a potent MOR-agonist carfentanil [55]. It is tempting to speculate that higher doses of opioid antagonists might be needed to sufficiently block the endogenous opioid system and allow latent sensitization to become apparent.

### Extent of Injury

The animal studies of latent sensitization with the plantar incision [17] and CFA [18], [19] model, induce deep tissue inflammation. These models are likely associated with an increased degree of nociception compared to the superficial BI-model, which may be inadequate for generating latent sensitization. There are no studies examining the severity of the primary injury and the latent sensitization. However, Maihöfner and co-workers showed activity in the pre-frontal cortex, secondary somatosensory cortex, insular cortex, anterior cingulate cortex and thalamus after repeated minor heat stimulation both at 46.7±0.4°C and 43.5±0.5_°C, (9 cm^2^, 15 s, left volar arm) [56] and in a different study at 46.7°C±2.0°C and 40.4°C±1.9°C (9 cm^2^, 24 s, left volar arm) [57], observations suggesting that even mild heat stimuli are processed by rostral neural centers. There is evidence that pain can induce changes in neuronal network connectivity and in chronic pain patients structural brain changes may occur [58].

### Time from Injury to Attempted Reinstatement

The interval between injury and assessment of latent sensitization in the experimental animal studies has been 21 days [17]–[19]. In the present study, due to the more superficial inflammatory injury, an interval of 3 days was used. Although no systematic research has been made in regard to the minimal necessary interval needed to show latent sensitization, it is possible that evaluating a different interval between the injury and testing could demonstrate latent sensitization.

### Methodological Issues

#### Assessment of secondary hyperalgesia areas

In the present study, areas of secondary hyperalgesia areas were assessed using a polyamide monofilament (nominal value 18, bending force of 890 mN). This is a relatively large monofilament, which may allow a more accurate assessment of hyperalgesia areas, when compared to smaller monofilaments of 200–300 mN [59]. These smaller monofilaments probably delineate much larger areas of hyperesthesia and allodynia, but not hyperalgesia [59].

In the rodent studies [18], [19] mechanical hypersensitivity was assessed by thresholds to monofilament stimulation, while in the present study changes in mechanical hypersensitivity were evaluated by pin-prick assessments of secondary hyperalgesia areas. These grading methods of hypersensitivity are clearly different, i.e. one method measures thresholds while the other measures areas. However, in humans the methods are inversely interrelated: increased sensitivity in the secondary hyperalgesia area, following a burn injury, is associated with a proportional decrease in mechanical pain thresholds and an increase in secondary hyperalgesia areas [60]–[62].

A methodological advantage of the present study was that 2 separate methods of secondary hyperalgesia area assessments, i.e. the BI- and BTS-methods, were used. Changes in hyperalgesia area after naloxone or placebo administration showed a high degree of agreement between the two methods; the same direction of change was observed in 16 out of 22 volunteers. However, it should be emphasized that the two methods differ in regard to induction of secondary hyperalgesia areas: in the BI-method re-instatement of secondary hyperalgesia following resolution of an injury was examined and with the BTS-method the response to an acute noxious stimulus was analysed.

#### Habituation

Habituation effects between the first BI (Day 1) and the second BI (Day 3), was seen in regard to secondary hyperalgesia areas, but not in regard to other variables tested. This effect has been reported before and thus was expected [35], [37]. However, any confounding is minimized by the randomization and the cross-over design: results were similar regardless of whether the volunteers were first given naloxone or placebo. However, a longer interval between sessions might reduce any habituation effect.

### Species Issue

Species differences may be such that the phenomenon has a different underlying mechanism or is expressed differently. No systematic research has directly compared latent sensitization between humans and rodents. The models of hyperalgesia and endpoints determined are quite different between the current study and previous rodent studies. For example, while we evaluated tactile hyperalgesia (response to pin) following a mild burn injury, previous animal studies evaluated tactile allodynia (response to von Frey hairs) following injection of an inflammogen (Corder et al) [18], [19] or incision plus opioid (Campillo et al) [17], [63]. Additional studies in animal models of mild burn injury are required to determine whether the parameters used in the current study are sufficient to induce latent sensitization in animals.

### Conclusion

In conclusion, although recent animal studies, based on an inflammatory injury, have shown a late re-instatement of secondary hyperalgesia following administration of an opioid-antagonist, the present study could not reproduce these results in a human first-degree burn injury model. The negative results might be explained by use of a low dose of naloxone (leading to an insufficient blockade of endogenous opioid receptors); the limited tissue injury by the model; incorrect timing of assessments relative to drug administration; or to species differences. Further studies are needed to fully examine the possibility of latent sensitization after injury in humans.
